# Parkin Overexpression Attenuates Sepsis-Induced Muscle Wasting

**DOI:** 10.3390/cells9061454

**Published:** 2020-06-11

**Authors:** Jean-Philippe Leduc-Gaudet, Dominique Mayaki, Olivier Reynaud, Felipe E. Broering, Tomer J. Chaffer, Sabah N. A. Hussain, Gilles Gouspillou

**Affiliations:** 1 Meakins-Christie Laboratories and Translational Research in Respiratory Diseases Program, Research Institute of the McGill University Health Centre; Department of Critical Care, McGill University Health Centre, Montréal, QC H4A 3J1, Canada; jean-philippe.leduc-gaudet@mail.mcgill.ca (J.-P.L.-G.); dominique.mayaki@muhc.mcgill.ca (D.M.); felipe.broering@mail.mcgill.ca (F.E.B.); jordichaffer@gmail.com (T.J.C.); 2 Division of Experimental Medicine, McGill University, Montréal, QC H4A 3J1, Canada; oreynaud26@gmail.com; 3 Département des sciences de l’activité physique, Faculté des Sciences, UQAM, Montréal, QC H2X 1Y4, Canada; 4 Groupe de recherche en Activité Physique Adaptée, Montréal, QC H2X 1Y4, Canada; 5 Département des sciences biologiques, Faculté des Sciences, UQAM, Montréal, QC H2X 1Y4, Canada; 6 Centre de Recherche de l’Institut Universitaire de Gériatrie de Montréal, Montréal, QC H3W 1W5, Canada

**Keywords:** muscle atrophy, septicemia, mitochondria, mitochondrial fusion, mitochondrial fission

## Abstract

Sepsis elicits skeletal muscle weakness and fiber atrophy. The accumulation of injured mitochondria and depressed mitochondrial functions are considered as important triggers of sepsis-induced muscle atrophy. It is unclear whether mitochondrial dysfunctions in septic muscles are due to the inadequate activation of quality control processes. We hypothesized that overexpressing Parkin, a protein responsible for the recycling of dysfunctional mitochondria by the autophagy pathway (mitophagy), would confer protection against sepsis-induced muscle atrophy by improving mitochondrial quality and content. Parkin was overexpressed for four weeks in the limb muscles of four-week old mice using intramuscular injections of adeno-associated viruses (AAVs). The cecal ligation and perforation (CLP) procedure was used to induce sepsis. Sham operated animals were used as controls. All animals were studied for 48 h post CLP. Sepsis resulted in major body weight loss and myofiber atrophy. Parkin overexpression prevented myofiber atrophy in CLP mice. Quantitative two-dimensional transmission electron microscopy revealed that sepsis is associated with the accumulation of enlarged and complex mitochondria, an effect which was attenuated by Parkin overexpression. Parkin overexpression also prevented a sepsis-induced decrease in the content of mitochondrial subunits of NADH dehydrogenase and cytochrome C oxidase. We conclude that Parkin overexpression prevents sepsis-induced skeletal muscle atrophy, likely by improving mitochondrial quality and contents.

## 1. Introduction

Sepsis is a complex syndrome characterized by an overwhelming infection that results in a severe systemic inflammatory response. Sepsis causes diverse vascular, metabolic and endocrine abnormalities that lead to multiple organ failure, and often result in death [1]. Amongst the very deleterious effects of sepsis is severe weakness, which involves both respiratory and limb skeletal muscles [2,3,4,5]. In the short term, sepsis-induced respiratory muscle weakness leads to difficulty removing patients from mechanical ventilation, increases the risk of the recurrence of respiratory failure, prolonged hospitalization and increased mortality [6]. In sepsis survivors discharged from the intensive care unit, the long-term ramifications of sepsis-induced limb muscle weakness included functional impairment, limited physical activity and poor quality of life [7].

There is currently a lack of effective therapies to either prevent or treat sepsis-induced skeletal muscle weakness, due in large part to the fact that its molecular and cellular bases are poorly understood. However, one clue lies at the ultrastructural level, where significant accumulations of damaged and dysfunctional mitochondria are characteristic of sepsis-induced muscle dysfunction [8,9]. Indeed, Bready et al. showed that, in human skeletal muscle, sepsis results in decreased complex I activity (a key enzyme of the mitochondrial electron transfer system) and declined the ATP/ADP ratio in skeletal muscles [10]. These defects in muscle bioenergetics were also observed in a rat model of sepsis [11]. By studying biopsies obtained from septic patients, Fredriksson K et al. described a 30% decrease in complex IV activity in limb skeletal muscles [12]. Several studies on experimental animals also reported that sepsis results in decreased mitochondrial respiration [13,14,15,16] and an increase in the mitochondrial production of reactive oxygen species (ROS) in skeletal muscle [17,18]. Sepsis has also been shown to increase the levels of morphologically abnormal mitochondria, such as those with disorganized cristae, translucent vacuoles and even myelin-like structures [13,19,20,21]. Recently, Owen et al. showed that persistent muscle weakness in mice that have survived sepsis is associated with abnormal mitochondrial ultrastructure, decreased respiration, decreased activity of complexes of the mitochondrial electron transfer system and persistent oxidative damage to muscle proteins [21].

In healthy muscles, damaged or dysfunctional mitochondria are selectively recycled in a process, known as mitophagy (selective autophagy of mitochondria), which is primarily regulated through the PINK1-Parkin pathway. Parkin, an E3 ubiquitin ligase encoded by the *Park2* gene, is a 465 amino acid protein that translocates to depolarized mitochondria to initiate mitophagy. Parkin-dependent mitophagy is regulated by PTEN-induced kinase 1 (PINK1), which acts upstream from Parkin. In healthy mitochondria, PINK1 is imported into the inner mitochondrial membrane and cleaved by PARL [22]. Cleaved PINK1 is then released into the cytosol where it is degraded by the proteasome system. In depolarized mitochondria, the importation of PINK1 into the inner mitochondrial membrane is blocked. PINK1 is no longer degraded and becomes phosphorylated and stabilized on the outer mitochondrial membrane [23,24,25,26]. Phosphorylated PINK1 triggers the recruitment of Parkin to the mitochondria. Parkin then ubiquitinates outer mitochondrial membrane proteins, including the fusion proteins MFN1, MFN2, MIRO and TOMM20 [27]. The degradation of MFN1 and MFN2 triggers mitochondrial fission and fragmentation, both of which are important to the recycling of mitochondria by the mitophagy pathway [28]. The functional importance of the PINK1-Parkin mitophagy pathway in regulating skeletal muscle mitochondrial function and quality in sepsis remains unknown. Recently, we reported that the genetic deletion of Parkin leads to the poor recovery of cardiac function in septic mice and increased sepsis-induced mitochondrial dysfunction in the heart [29]. We also demonstrated that autophagy is significantly induced in the skeletal muscles of septic mice and that the induction of autophagy is associated with increased muscle Parkin levels, suggesting that mitophagy was induced [20,30]. However, several morphologically and functionally abnormal mitochondria were observed in the electron micrographs of septic muscles, indicating that the mitophagy that was induced was likely insufficient to the task of completely recycling defective mitochondria [20,30]. Based on this reasoning, we hypothesized that enhancing mitophagy through Parkin overexpression would attenuate the impact of sepsis on skeletal muscles and their mitochondria. To test this hypothesis, Parkin was overexpressed for four weeks in the skeletal muscles of young mice using intramuscular injections of adeno-associated viruses (AAVs). The cecal ligation and perforation (CLP) procedure, a widely used model of sepsis [31], was used to induce sepsis. Sham-operated animals served as controls. We found that Parkin overexpression prevents sepsis-induced mitochondrial morphological injury and reverses the decline in mitochondrial protein content. We also found that Parkin overexpression protects against sepsis-induced myofiber atrophy. These findings indicate that defective mitophagy in sepsis can be therapeutically manipulated as a means of counteracting sepsis-induced muscle dysfunction.

## 2. Materials and Methods

### 2.1. Animal Procedures

All experiments were approved (#2014-7549) by the Research Ethics Board of the Research Institute of the McGill University Health Centre (MUHC-RI) and are in accordance with the principles outlined by the Canadian Council of Animal Care. Three-week-old male wild-type C57BL/6J mice (Charles River Laboratories, Saint-Constant, QC, Canada) were used for our experiments. All mice were group-housed under a standard 12:12 h light/dark cycle with food and water available ad libitum.

### 2.2. AAV Injections in Skeletal Muscle

All of the adeno-associated viruses (AAVs) used in our experiments were purchased from Vector Biolabs (Malvern, PA, USA) and were of Serotype 1, a serotype highly effective in transducing skeletal muscle cells [32]. Four-week-old mice were first anesthetized with an isoflurane (2.5 to 3.5%), and AAV1s containing a muscle specific promoter (muscle creatine kinase), a sequence coding for the reporter protein GFP and a sequence coding for Parkin (details on the AAV1 construction are available in Supplementary Figure S1) were then intramuscularly injected (25 µL per site; 1.5 × 10^11^ gc) into the gastrocnemius (GAS) muscles in the right leg. In this AAV1 construction, the sequences coding for Parkin and GFP were separated by a sequence coding for the auto-cleavable 2A peptide, allowing for the separation of the Parkin and GFP proteins once translated. Control AAV1s containing only the GFP sequence under the control of the MCK promoter were injected into the contralateral leg. Because the AAV1 recombination site in the wild-type AAV1s was deleted in these recombined AAV1s, both GFP and Parkin expression comprised episomal expression without integration into the host DNA.

### 2.3. Cecal Ligation and Perforation

After four weeks of AAV1 injection, the mice were subjected to cecal ligation and perforation or sham surgery. The cecal ligation and puncture (CLP) model, which closely mimics the clinical features of human sepsis [31], was performed to induce polymicrobial sepsis as described previously [30,33] with minor modifications. Briefly, the mice were first anesthetized with isoflurane (~3%; Piramal Critical Care). A midline abdominal incision (~2 cm) was then performed. The cecum was carefully ligated at ~1 cm from its distal portion. The ligated cecum was perforated by a through-and-through puncture performed with 25^1/2^ gauge needle in a sterile environment. Next, the ligated cecum was gently compressed to extrude a small amount of the cecal contents through the punctured holes. The cecum was then replaced in the abdominal cavity. The peritoneum was then closed in two separate layers using 3–0 absorbable polyfilament interrupted sutures. The skin was finally closed with a surgical staple (9 mm AutoClip^®^ System, Fine Scientific tools, North Vancouver, BC, Canada). All of the animals received subcutaneous injections of buprenorphine (0.05 to 0.2 mg/kg in 1 mL of 0.9% saline) immediately after surgery. To minimize pain, buprenorphine was administered every 12 h (0.05 mg/kg in ~100 µL of 0.9% saline). The sham-operated mice were subjected to identical procedures with the exception of the cecum ligation and puncture. All of the animals were closely monitored for signs of excessive pain or distress, such as lack of movement, agonal breathing or excessive body loss (20%), by investigators and by the vivarium staff from the IR-MUHC. Any mouse reaching endpoint criteria was immediately euthanized.

### 2.4. Tissue Collection

Mice were anesthetized with isoflurane and subsequently euthanized by cervical dislocation 48 h after sham or CLP procedures. The gastrocnemius (GAS) muscles were carefully removed from both legs and cut in half; one half was mounted for histology and small strips were prepared for transmission electron microscope (TEM) analyses, as previously described [34]. The rest of the GAS was quickly frozen in liquid nitrogen and stored −80 °C until use for immunoblotting and qPCR experiments.

### 2.5. Fiber Size Determination

Muscles samples were mounted on plastic blocks in tragacanth gum and frozen in liquid isopentane cooled in liquid nitrogen. The samples were then stored until use at −80 °C. The samples were cut into 10 µm cross-sections using a cryostat (Leica Biosystem Inc., Concord, ON, Canada) at −20 °C and then mounted on lysine coated slides (Superfrost) to assess muscle fiber size, as described in [32,34]. To this end, the muscle cross-sections were first allowed to reach room temperature and were rehydrated with phosphate buffered saline (PBS, pH 7.2) and then blocked with goat serum (10% in PBS). The sections were then incubated with primary rabbit IgG polyclonal anti-laminin antibody (MilliporeSigma, Oakville, ON, Canada, L9393, 1:750) for 1 h at room temperature. The sections were then washed three times in PBS before being incubated for 1 h at room temperature with an Alexa Fluor 594 goat anti-rabbit IgG antibody (Invitrogen, Burlington, ON, Canada A-11037, 1:500). The sections were then washed three times in PBS and the slides were cover-slipped using Prolong Gold (Invitrogen, P36930) as mounting medium. The slides were imaged with a Zeiss Axio Imager 2 fluorescence microscope (Zeiss, Dorval, QC, Canada). The median minimum Feret’s diameter of the muscle fibers, a reliable marker of myofiber size [35], was determined for each muscle sample using at least 200 fibers per muscle sample (average number ± SD of fiber analyzed for each group: sham AAV-GFP, 317 ± 62; sham AAV-Parkin, 300 ± 15; CLP AAV-GFP, 345 ± 30; CLP AAV-Parkin, 304 ± 46). Analyses were performed using ImageJ (NIH, Bethesda, MD, USA, https://imagej.nih.gov/ij/).

### 2.6. Transmission Electron Microscopy (TEM)

The samples for TEM were prepared as described in [34,36,37]. Briefly, small strips prepared from GAS were incubated in 2% glutaraldehyde buffer solution in 0.1 M cacodylate (pH 7.4) and were subsequently post-fixed in 1% osmium tetroxide in 0.1 M cacodylate buffer. Tissues were then dehydrated via increasing the concentrations of methanol to propylene oxide and infiltrated and embedded in EPON^TM^ resins at the Facility for Electron Microscopy Research (FEMR) of McGill University. Ultrathin sections (60 nm) were cut longitudinally using an ultramicrotome (Ultracut III, Reichert-Jung, Leica Biosystem Inc., Concord, ON, Canada) and mounted on nickel carbon-formvar-coated grids for electron microscopy. Uranyl acetate and lead citrate stained sections were then imaged using a FEI Tecnai 12 transmission electron microscope at 120 kV, and images were digitally captured using a XR80C CCD camera system (AMT, Woburn, MA, USA) at a magnification of 1400×. Individual intermyofibrillar (IMF) mitochondria from all groups were manually traced in longitudinal orientations using ImageJ 2.0.0 software (NIH, Bethesda, MD, USA, https://imagej.nih.gov/ij/) to measure the following morphological characteristics: area (in μm^2^), perimeter (μm), circularity (4π·(surface area/perimeter^2^)), Feret’s diameter (longest distance (μm) between any two points within a given mitochondrion), aspect ratio (major axis/minor axis)—a measure of the “length to width ratio” and form factor (perimeter/4π·surface area)—a measure sensitive to the complexity and branching aspect of mitochondria [34,36,37]. An index of mitochondrial morphological complexity was finally calculated as follows: Mitochondrial complexity index = Aspect ratio × Form Factor. Details on the number of IMF mitochondria that were traced are available in the figure legends.

### 2.7. Immunoblotting

Frozen skeletal muscle tissues (15–30 mg) were homogenized in an ice-cold lysis buffer (50 mM Hepes, 150 mM NaCl, 100mM NaF, 5 mM EDTA, 0.5% Triton X-100, 0.1 mM DTT, 2 µg/mL leupeptin, 100 µg/mL PMSF, 2 µg/mL aprotinin, and 1 mg/100 mL pepstatin A, pH 7.2) using Mini-beadbeater (BioSpec Products) with a ceramic bead at 60 Hz. The muscle homogenates were kept on ice for 30 min with periodic agitation and were then centrifuged at 5000 *g* for 15 min at 4 °C, the supernatants were collected, and the pellets were discarded. The protein contents in each sample were determined using the Bradford method. The aliquots of crude muscle homogenates were mixed with Laemmli buffer (6×, reducing buffer, # BP111R, Boston BioProducts, Ashland, MA, USA) and subsequently denatured for 5 min at 95 °C. Equal amounts of protein extracts (30 µg per lanes) were separated by SDS-PAGE, and then transferred onto polyvinylidene difluoride (PVDF) membranes (Bio-Rad Laboratories, Saint-Laurent, QC, Canada) using a wet transfer technique. The total proteins on the membranes were detected with Ponceau-S solution (MilliporeSigma #P3504). The membranes were blocked in PBS + 1% Tween^®^ 20 + 5% bovine serum albumin (BSA) for 1 h at room temperature and then incubated with the specific primary antibodies overnight at 4 °C. The complete list of antibodies used for immunoblots analysis can be found in Supplementary Table S1. Membranes were washed in PBST (3 × 5 min) and incubated with HRP-conjugated secondary anti-rabbit or anti-mouse secondary antibodies (Abcam, Toronto, ON, Canada, cat# Ab6728, Ab6721) for 1 h at room temperature, before further washing in PBST (3 × 5 min). Immunoreactivity was detected using an enhanced chemiluminescence substrate (Pierce™, Thermo Fisher Scientific, Saint-Laurent, QC, Canada) with the ChemiDoc™ XRS+ Imaging System. The optical densities (OD) of the protein bands were quantified using ImageLab software (Bio-Rad Laboratories) and normalized to loading control (Ponceau-stained PVDF membranes). Immunoblotting data are expressed as relative to Sham AAV-GFP.

### 2.8. Quantitative Real-Time PCR

Total RNA was extracted from frozen muscle samples using a PureLink™ RNA Mini Kit (Invitrogen Canada, Burlington, ON, Canada). The quantification and purity of RNA was assessed using the A260/A280 absorption method. Total RNA (2 μg) was reverse transcribed using a Superscript II^®^ Reverse Transcriptase Kit and random primers (Invitrogen, Burlington, ON, Canada). The reactions were incubated at 42 °C for 50 min and at 90 °C for 5 min. The real-time PCR detection of mRNA expression was performed using a Prism^®^ (Graphpad, San Diego, CA, USA) 7000 Sequence Detection System (Applied Biosystems, Foster, CA, USA). The cycle threshold (C_T_) values were obtained for each target gene. The ΔC_T_ values (normalized gene expression) were calculated as C_T_ of target gene minus C_T_ of the geometric means of three housekeeping genes (*Cyclophilin B*, *β-Actin* and *18S*). The relative mRNA level quantifications of target genes were determined using the threshold cycle (ΔΔC_T_) method, as compared to sham AAV-GFP. The primer sequences for all genes are found in Supplementary Table S2.

### 2.9. Data Analysis and Statistics

All statistical analyses were performed using GraphPad Prism 8 (GraphPad, San Diego, CA, USA). Comparisons of initial body weight and body weight loss between sham-operated and CLP mice were performed using unpaired bilateral student *t*-tests (*p*-values < 0.05 were considered statistically significant). Comparisons of the effects of Parkin overexpression on parameters of interest were performed using two-way repeated measures analysis of variance (ANOVA) (except for comparisons of mitochondrial shape descriptors, as detailed below). Corrections for the multiple comparisons following two-way repeated measures ANOVA were performed with the two-stage step-up method of Benjamini and Krieger and Yekutieli (*q* < 0.1 was considered statistically significant). One-way ANOVA followed by the two-stage step-up method of Benjamini and Krieger and Yekutieli were used for the following comparisons: sham AAV-GFP vs. CLP AAV-GFP; sham AAV-GFP vs. CLP AAV-Parkin; sham AAV-Parkin vs. CLP AAV-GFP; sham AAV-Parkin vs. CLP AAV-Parkin (except for comparisons of mitochondrial shape descriptors, as detailed below) (*q* < 0.1 was considered statistically significant). Differences for the median values of shape descriptors to assess mitochondrial morphology were assessed using a Kruskal–Wallis test followed by a Dunn’s multiple comparisons test (adjusted *p*-values < 0.05 were considered statistically significant). The exact numbers of animals within each group in all figures are indicated in the figure legends.

## 3. Results

### 3.1. Successful Overexpression of Parkin in Skeletal Muscles of Sham and CLP Operated Mice

**Figure 1:**
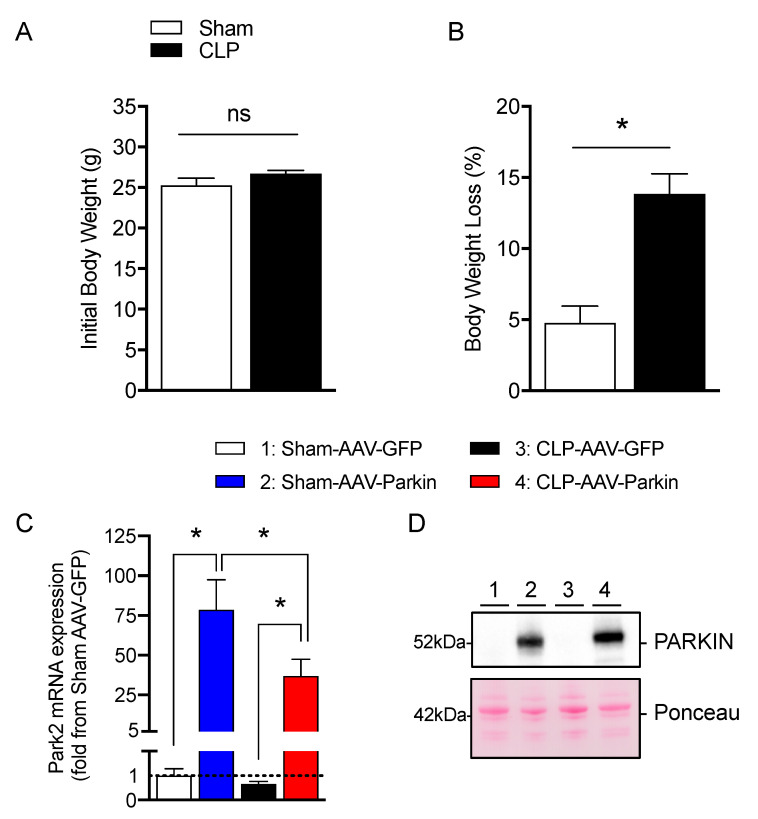
Effective Parkin overexpression in skeletal muscles of Sham and CLP operated mice. (**A**) Initial body weight and (**B**) percent of body weight loss in Sham-operated and or CLP mice. (**C**) qPCR analysis of *Park2* expression levels in the gastrocnemius muscles injected with either AAV-GFP or AAV-Parkin in Sham and CLP mice. (**D**) Representative Parkin immunoblots and its corresponding ponceau S stain performed on gastrocnemius samples of Sham and CLP mice injected with either AAV-GFP or AAV-Parkin. 1 = Sham-AAV-GFP; 2 = Sham-AAV-Parkin; 3 = CLP-AAV-GFP; 4 = CLP-AAV-Parkin. Data are presented as mean ± SEM (*n* = 7–9/group; * = statistically significant; ns = not statistically significant).

Four weeks after the intramuscular injections of AAVs, mice were subjected to cecal ligation and perforation (CLP) to induced polymicrobial sepsis. Sham-operated mice were used as control. At baseline (prior to sham and CLP procedures), body weight values were similar in the sham and CLP groups, as shown in Figure 1A. Body weight loss was more pronounced in the CLP group relative to the sham group (−13.8 ± 1.4% vs. −4.7±1.1%, respectively, *p* < 0.05), as shown in Figure 1B. As shown in Figure 1C,D, the intramuscular injection of AAV-Parkin significantly increased *Park2* mRNA expression and Parkin protein content, in the skeletal muscles of both Sham-operated and CLP mice. These results demonstrate that our approach was successful in overexpressing Parkin in mouse skeletal muscle.

### 3.2. Parkin Overexpression Attenuates Sepsis-Induced Skeletal Muscle Atrophy

**Figure 2:**
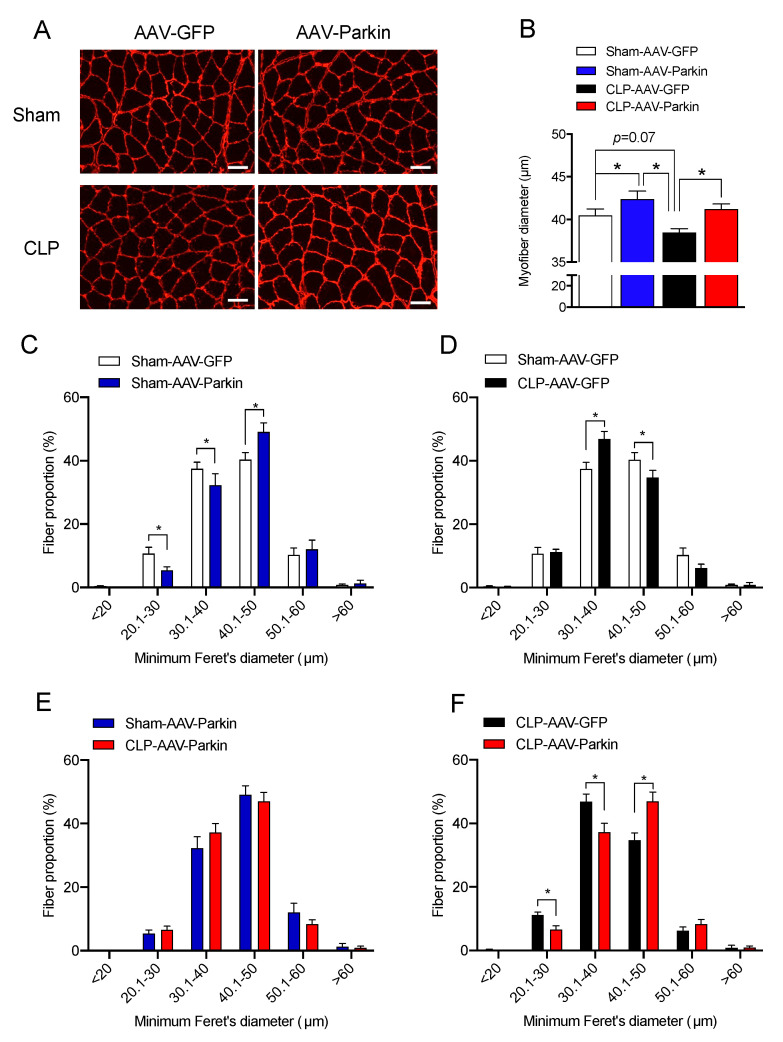
The impact of Parkin overexpression and sepsis on skeletal muscle fiber size. (**A**) Representative gastrocnemius (GAS) cryosections stained for laminin in all experimental groups. Scale bar: 50µm. (**B**) Quantification of minimum Ferret diameter of GAS myofibers of Sham and CLP animals injected with either AAV-GFP or AAV-Parkin. (**C**) Minimum Ferret distribution of the GAS myofibers of Sham AAV-GFP (*n* = 8 mice; 316 ± 21 fibers per GAS were traced) vs. Sham AAV-Parkin (*n* = 8 mice; 300 ± 5 fibers per GAS were traced). (**D**) Minimum Ferret distribution of the GAS myofibers of Sham AAV-GFP (*n* = 8 mice; 316 ± 21 fibers per GAS were traced) vs. CLP AAV-GFP (*n* = 6 mice; 345 ± fibers per GAS were traced). (**E**) Minimum Ferret distribution of the GAS myofibers of Sham AAV-Parkin (*n* = 8 mice; 300 ± 5 fibers per GAS were traced) vs. CLP AAV-Parkin (*n* = 6 mice; 304 ± 18 fibers per GAS were traced). (**F**) Minimum Ferret distribution of the GAS myofibers of CLP AAV-GFP (*n* = 6 mice; 345 ± fibers per GAS were traced) vs. CLP AAV-Parkin (*n* = 6 mice; 304 ± 18 fibers per GAS were traced). Data are presented as mean ± SEM. (*n* = 6–8/group; * = statistically significant).

The effect of Parkin overexpression on muscle fiber size was evaluated 48 h after CLP, based on our previous reports which revealed that limb muscle atrophy develops at this time point [30,33]. In the sham group, Parkin expressing muscles had larger myofiber diameters relative to those expressing GFP, as shown in Figure 2B,C. This observation is in line with our previous report [32]. In the CLP group, GFP expressing muscles displayed a trend towards smaller myofiber diameters and a decreased proportion of large fibers relative to those expressing GFP in the sham group, as shown in Figure 2B,D, all of which are indicative of myofiber atrophy. As shown Figure 2B,E, no sign of atrophy was detected in the Parkin overexpressing muscles of CLP mice when compared to the Parkin expressing muscles of Sham-operated mice. In addition, the Parkin overexpressing muscles of CLP mice displayed larger myofibers vs. the GFP expressing muscles of CLP mice, as shown in Figure 2B,F. These results indicate that Parkin overexpression prevented the development of muscle atrophy in the CLP group and increased muscle fiber diameter in the sham group.

### 3.3. The Impact of Parkin Overexpression and Sepsis on Skeletal Muscle Catabolic Signaling

**Figure 3:**
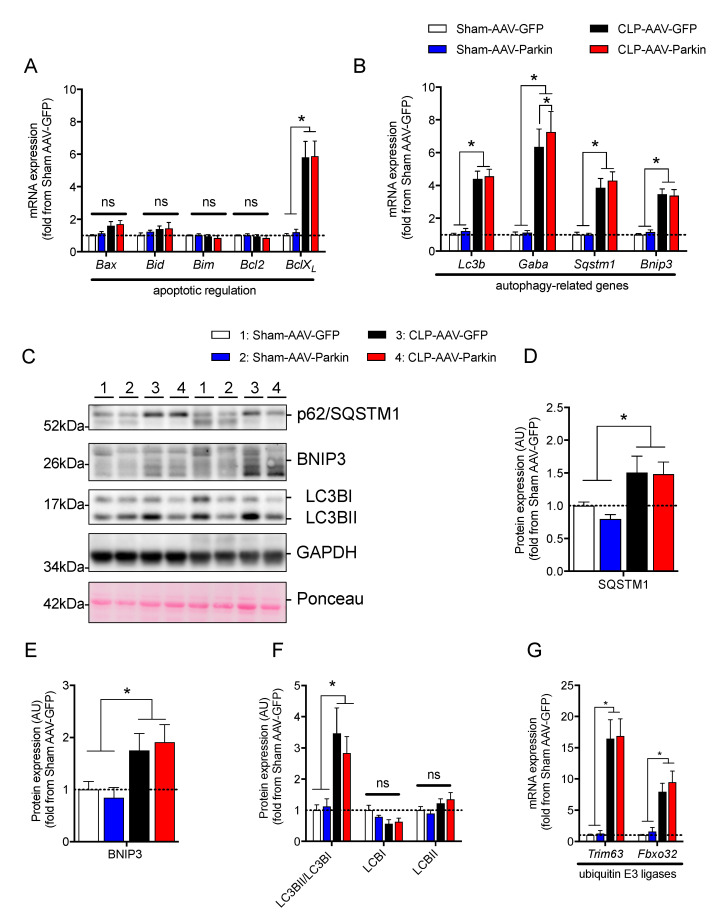
The impact of Parkin overexpression and sepsis on skeletal muscle catabolic signaling. (**A**) qPCR analysis of the mRNA expression of genes regulating apoptosis in the gastrocnemius (GAS) muscles of Sham and CLP animals injected with either AAV-GFP or AAV-Parkin. (**B**) qPCR analysis of autophagy-related gene expression in the gastrocnemius (GAS) muscles of Sham and CLP animals injected with either AAV-GFP or AAV-Parkin. *Gaba.* refers to *Gabarapl1*. (**C**) Immunoblot detection of SQSMT1(p62), BNIP3, LC3I/LC3II and GAPDH. (**D**) Quantification of SQSMT1 (p62) content. (**E**) Quantification of BNIP3 protein content. (**F**) Quantification of LC3I and LC3II protein content, as well as the LC3II to LC3I ratio. (**G**) qPCR analysis of *Fbxo32* (Atrogin-1) and *Trim63* (MuRF1) gene expression levels in the GAS muscles of Sham and CLP animals injected with either AAV-GFP or AAV-Parkin. 1 = Sham-AAV-GFP; 2 = Sham-AAV-Parkin; 3 = CLP-AAV-GFP; 4 = CLP-AAV-Parkin. Data are presented as mean ± SEM. (*n* = 6–9/group, * = statistically significant; ns = not statistically significant).

We then investigated whether Parkin overexpression and sepsis affect the expression levels of apoptotic and autophagy-related genes. Neither Parkin overexpression nor sepsis affected the mRNA levels of pro-apoptotic *Bax*, *Bid*, *Bim*, and anti-apoptotic *Bcl2*, depicted in Figure 3A. As shown in Figure 3A, the expression level of BclXL was higher in septic animals. qPCR analyses revealed a significant increase in the mRNA levels of *Lc3b*, *Sqstm1*, *Gabarapl* and *Bnip3* in septic mice, shown in Figure 3B. The expression of *Gabarapl* was significantly higher in the Parkin overexpressing muscles of septic animals. No other impacts of Parkin overexpression on apoptotic and autophagy-related genes were observed. In line with our gene expression data, the protein contents of SQSTM1 (also known as p62) and BNIP3, two proteins regulating autophagy and mitophagy, were increased in septic animals, as shown in Figure 3C–E. The ratio of LC3-II to LC3-I was significantly increased in septic mice, suggesting an induction of autophagy, shown in Figure 3F. No impact of Parkin overexpression on the content of SQSTM1 and BNIP3 and the LC3-II to LC3-I ratio could be evidenced. We then assessed the expression levels of two key E3 ligases known to contribute to skeletal muscle atrophy [38,39], Fbxo32 (Atrogin-1) and Trim63 (MuRF1). The expression of these two E3 ligases was significantly increased in the skeletal muscle of septic animals, shown in Figure 3G. Parkin overexpression did not impact Fbxo32 and Trim63 expression. It is worth mentioning that neither Parkin overexpression nor sepsis had an impact on the content or phosphorylation levels of AKT and 4EBP1, two key proteins involved in the regulation of protein synthesis, as shown in Supplementary Figure S2. Taken altogether, these data indicate that Parkin overexpression did not attenuate sepsis-induced increases in catabolic signaling.

### 3.4. The Impact of Parkin Overexpression and Sepsis on the Expression of Genes and Proteins Regulating Mitochondrial Biology

**Figure 4:**
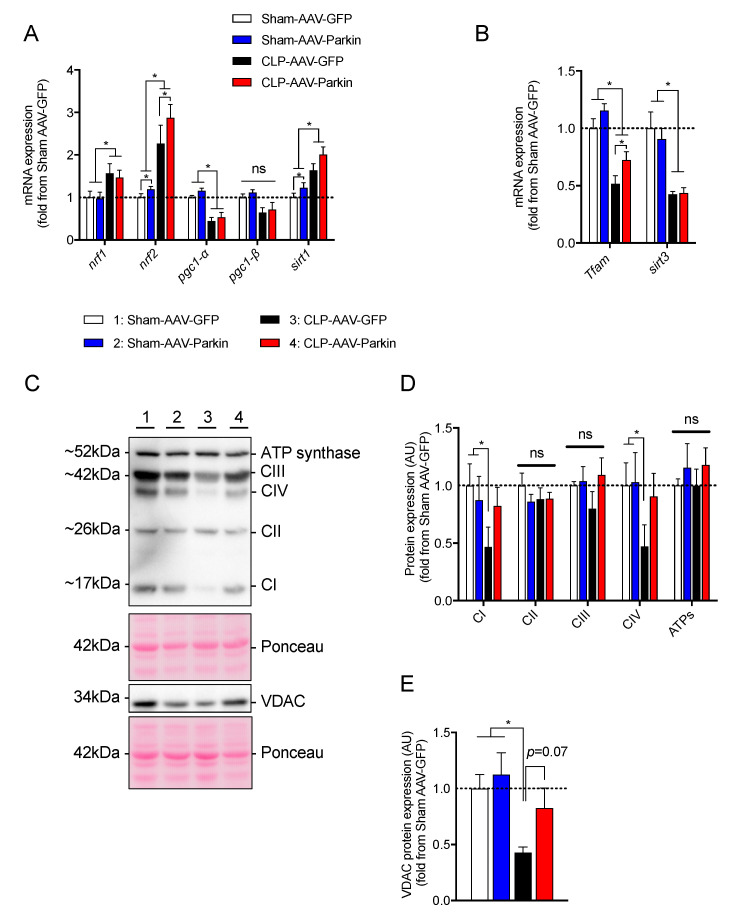
The impact of Parkin overexpression and sepsis in skeletal muscle on genes regulating mitochondrial biogenesis and on mitochondrial protein contents. (**A**,**B**) qPCR analysis of genes involved in mitochondrial biology. (**C**) Representative immunoblots performed with primary antibodies against representative subunits of the OXPHOS complexes and VDAC. Ponceau stains were used as loading controls. (**D**,**E**) Quantification of the contents of (**D**) representative subunits of the OXPHOS complexes and (**E**) VDAC. 1 = Sham-AAV-GFP; 2 = Sham-AAV-Parkin; 3 = CLP-AAV-GFP; 4 = CLP-AAV-Parkin. Data are presented as mean ± SEM. (*n* = 6–9/group, * = statistically significant; ns = not statistically significant).

Since Parkin plays a key role in mitochondrial quality control [23,24,25,26,40], and because sepsis is well known to impair mitochondrial function, we investigated whether Parkin overexpression could attenuate the impact of sepsis on skeletal muscle mitochondria. To this end, we first quantified the expression levels of the key transcriptional regulators of mitochondrial biology. As shown in Figure 4A,B, sepsis resulted in an increase in *Nrf1, Nrf2,* and *Sirt1* mRNA expression levels. In contrast, sepsis resulted in a decrease in the expression of *Pgc1-α, Tfam and Sirt3*, as shown in Figure 4A,B. In the skeletal muscles of both Sham-operated and CLP mice, Parkin overexpression resulted in a significant increase in *Nrf2* mRNA expression, depicted in Figure 4A. Parkin overexpression also led to an increased expression of *Sirt1* in the muscles of Sham-operated mice and an increase in *Tfam* expression in the muscles of CLP mice, as shown in Figure 4B.

We next assessed the impact of sepsis and Parkin overexpression on the content of proteins of the mitochondrial oxidative phosphorylation (OXPHOS) system. As shown in Figure 4D, sepsis significantly decreased the content of the representative subunits of Complex I and Complex IV. This finding is consistent with previous reports, which documented decreased mitochondrial contents in septic skeletal muscles [10,12,13,14]. Similarly, sepsis lowered VDAC protein content by 58% in the GFP expressing skeletal muscles, as shown in Figure 4E. Importantly, no impact of sepsis was observed on Complex I, Complex IV and VDAC contents in the Parkin overexpressing muscles, as shown in Figure 4D,E. Taken together, these data strongly suggest that Parkin overexpression prevented the inhibitory effect of sepsis on muscle mitochondrial content.

### 3.5. Effects of Parkin Overexpression and Sepsis on Mitochondrial Morphology and Dynamics

**Figure 5:**
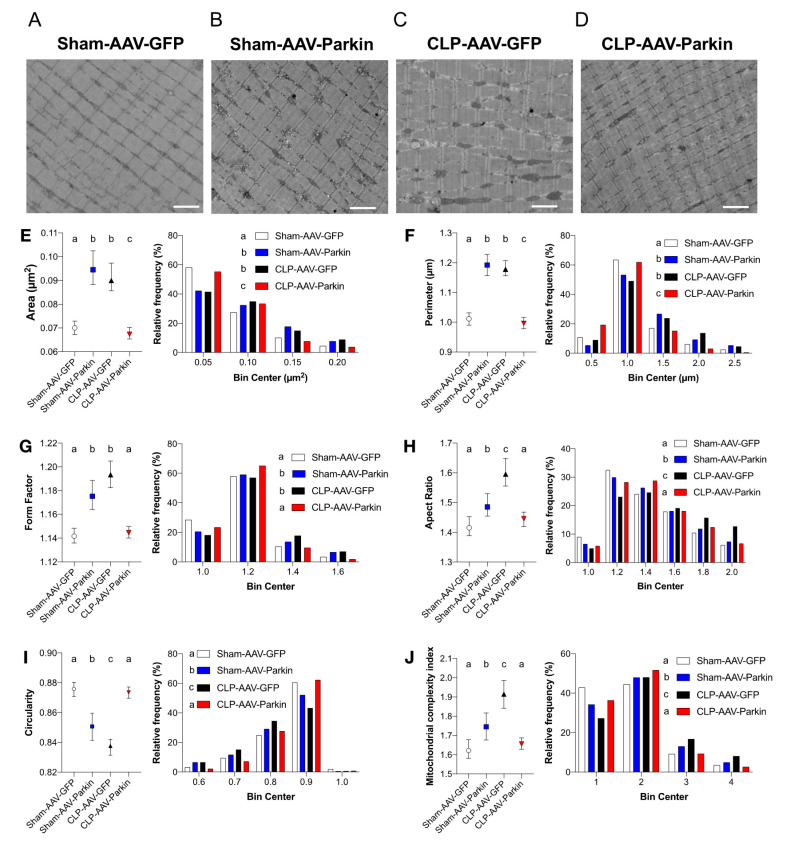
The impact of sepsis and Parkin overexpression on mitochondrial morphology in skeletal muscle. (**A**–**D**) Representative longitudinal TEM images from all groups that were used to assess mitochondrial morphology. Scale bar: 2µm. (**E**–**J**) Median values with 95% confidence interval (left) and relative frequencies (right) of multiple mitochondrial shape descriptors (Sham-AAV-GFP: *n* = 1246; Sham-AAV-Parkin: *n* = 728; CLP-AAV-GFP: *n* = 1149; CLP-AAV-Parkin: *n* = 1206). Groups not sharing a letter are significantly different (differences were tested using a Kruskal–Wallis test followed by a Dunn’s multiple comparisons test; *p* < 0.05).

To analyze the impact of sepsis and Parkin overexpression on skeletal muscle mitochondrial morphology, we used TEM to evaluate the morphology of intermyofibrillar (IMF) mitochondria of the GAS of sham and CLP mice. Representative TEM images obtained from the GAS of sham and CLP mice are shown in Figure 5A–D and Supplemental Figure S3. In the CLP group, IMF mitochondria of GFP expressing muscles were larger, less circular and more complex (i.e., higher values of aspect ratio and form factor) than IMF mitochondria of GFP expressing muscles of the sham group, shown in Figure 5E–J. In the sham group, Parkin overexpressing muscles had larger, less circular and more complex IMF mitochondria compared to GFP expressing muscles, as shown in Figure 5E–J. In the CLP group, Parkin overexpressing muscles had smaller, more circular and simpler IMF mitochondria compared to GFP expressing counterparts, shown in Figure 5 E–J. Taken together, these results indicate that sepsis results in enlarged and more complex mitochondria, an impact that is abolished by Parkin overexpression.

**Figure 6:**
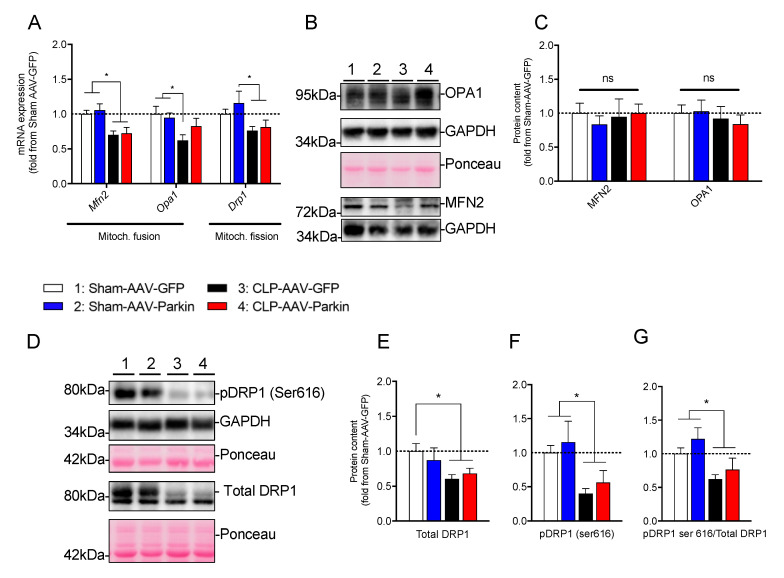
The impact of sepsis and Parkin overexpression on mitochondrial dynamics in skeletal muscle. (**A**) qPCR analysis of mitochondrial dynamic-related gene expression in the GAS muscles of Sham and CLP animals injected with either AAV-GFP or AAV-Parkin. (**B**) Representative immunoblots of OPA1, GADPH and MFN2. Ponceau stains or GAPDH immunoblots were used as loading controls. (**C**) Quantification of OPA1, GADPH and MFN2 content. (**D**) Representative immunoblots performed with primary antibodies against pDRP1(ser616) and total DRP1. Ponceau stains or GAPDH immunoblots were used as loading controls. (**E**) Quantification of DRP1 content. (**F**) Quantification of the contents of pDRP1(ser 616) content. (**G**) Quantification of the pDRP1(ser 616) to total DRP1 ratio. 1 = Sham-AAV-GFP; 2 = Sham-AAV-Parkin; 3 = CLP-AAV-GFP; 4 = CLP-AAV-Parkin. Data are presented as mean ± SEM. (*n* = 6–9/group; * = statistically significant; ns = not statistically significant).

To gain better insights into the mechanisms underlying the impact of sepsis and Parkin overexpression on mitochondrial morphology, we next assessed the expression and content of major genes and proteins regulating mitochondrial dynamics. In the Sham-operated mice, Parkin overexpression had no impact on the mRNA expression and protein levels of *Mfn2*, *Opa1* and *Drp1*, as shown in Figure 6A–G. As shown in Figure 6A, sepsis in GFP expressing muscles resulted in a decrease in the mRNA levels of pro-fusion *Mfn2* and *Opa1* and pro-fission *Drp1*. In Parkin overexpressing muscles, CLP resulted in a significant decrease in the mRNA levels of Mfn2 and Drp1, while Opa1 expression remained unaffected, shown in Figure 6A. At the protein level, no impact of sepsis or Parkin overexpression could be evidenced on MFN2 and OPA1 protein content. Interestingly, DRP1 protein levels were lower in the GFP and Parkin expressing muscles of CLP mice, relative to the sham group, as shown in Figure 6D,E. Similarly, DRP1 phosphorylation on Ser^616^, an activation site which triggers DRP1 translocation from the cytoplasm to mitochondria to promote mitochondrial fission [41], was also decreased in the GFP and Parkin expressing muscles of the CLP group, relative to the sham group, as shown in Figure 6D–G. These results indicate that sepsis seems to result in an inhibition of mitochondrial fission and that this effect was not influenced by Parkin overexpression.

## 4. Discussion

The accumulation of dysfunctional and injured mitochondria in skeletal muscles is believed to play a key role in the development of muscle weakness during sepsis [8,9]. In the current study, we investigated whether overexpressing Parkin, a key component of the PINK1-Parkin mitophagy pathway, could attenuate the negative impact of sepsis on skeletal muscles and their mitochondria. The current study indicates that Parkin overexpression prevented sepsis-induced accumulation of enlarged and complex mitochondria in the limb muscles of mice. Parkin overexpression also attenuated the sepsis-induced decrease in the content of complexes I and IV of the mitochondrial electron transfer system and prevented the development of limb muscle atrophy in septic mice. These results expand recent studies demonstrating that Parkin exerts protective effects on skeletal muscle health. Indeed, our group has recently reported that *Park2^-/-^* mice have decreased limb muscle contractility, depressed muscle mitochondrial respiration, increased mitochondrial uncoupling and enhanced susceptibility to the opening of mitochondrial permeability transition pore compared to wild-type (WT) mice [42]. *Park2^-/-^* mice also exhibit the impaired recovery of cardiac contractility and depressed cardiac mitochondrial functions in sepsis [29]. More recently, Peker et al. have reported that Parkin knockdown in C2C12 cells results in myotubular atrophy and that *Park2^-/-^* mice have decreased muscle mitochondrial respiration and increased levels of reactive oxygen species and fiber atrophy [43]. Parkin overexpression in the muscles of *Drosophila melanogaster* increased mitochondrial content, decreased proteotoxicity and extended lifespan [44]. Our finding that Parkin overexpression in the Sham group increased limb muscle fiber diameters is in accordance with our recent study documenting that Parkin overexpression for several months in young mice causes muscle hypertrophy, while in old mice, Parkin overexpression attenuates ageing-related loss of muscle mass and strength, increases mitochondrial content and enzymatic activities and protects from ageing-related oxidative stress, fibrosis and apoptosis [32]. Taken together, our current findings and published studies highlight the protective role that Parkin plays in skeletal muscle health.

Our findings that sepsis elicits distinct changes in skeletal muscle mitochondria, such as decreased VDAC level (a marker of mitochondrial content [45,46,47]), the downregulation of three mitochondrial biogenesis genes (*Pgc1-α*, *Tfam* and *Sirt3*) and decreased complexes I and IV levels, are in agreement with published studies on septic humans and experimental animals [10,12,13,14,20]. We report for the first time that Parkin overexpression in skeletal muscle prevents the inhibitory effects of sepsis on the expression of *Tfam* and on the content of complexes I and IV, as well as VDAC. Based on these results, we speculate that Parkin overexpression might have improved mitochondrial functions in septic muscles. This speculation is supported by the observation that Parkin overexpression increases mitochondrial content and enzymatic activities in normal skeletal muscles [32,44]. We should emphasize that, in the current study, Parkin overexpression increased *Nrf2* expression in the skeletal muscles of septic animals. Considering the role that this transcription factor has in the regulation of the expression of several anti-oxidant enzymes [48], we anticipate that increased *Nrf2* levels in muscles overexpressing Parkin might have contributed to the protection of mitochondrial morphology and contents in septic animals.

Mitochondria form a dynamic network constantly undergoing fusion and fission events that tightly regulate the shape (i.e., morphology), size and number of mitochondria [41,49]. In the present study, we show that sepsis significantly alters mitochondrial morphology by increasing the proportion of enlarged and more complex IMF mitochondria. These results extend previous observations, showing that sepsis causes major alterations of the mitochondrial ultrastructure in skeletal muscle [13,19,20,21]. This increase in mitochondrial size and complexity in septic muscles might have been caused by decreased DRP1 contents and activation [41], which are expected to alter the fusion/fission balance towards increased mitochondrial fusion. Since mitochondrial fission is required for mitochondrial degradation through mitophagy [50], it is possible that decreased DRP1 content and activation may play a role in the accumulation of damaged and dysfunctional mitochondria in septic muscles by impairing muscle capacity to recycle dysfunctional mitochondria through the mitophagy pathway. It should also be noted that a decrease in DRP1 content per se might have also played a role in myofiber atrophy. Indeed, a recent study showed that muscle-specific DRP1 deletion results in severe muscle dysfunction, characterized by atrophy, weakness, fiber degeneration and mitochondrial dysfunction [51]. Importantly, we found that Parkin overexpression attenuated sepsis-induced changes in mitochondrial morphology and rendered muscle mitochondria to be smaller, more circular and simpler, relative to muscles expressing GFP. These findings are in agreement with previous reports, indicating that Parkin overexpression in skeletal muscles and neurons stimulates mitochondrial fragmentation [44,52]. We speculate that the decrease in mitochondrial size and complexity in septic muscles overexpressing Parkin might have facilitated the recycling of damaged/dysfunctional mitochondria.

A puzzling result of the present study is the increase in the proportion of enlarged and more complex mitochondria in the Parkin overexpressing muscles of sham-operated mice. The mechanisms behind the differences in the effects of Parkin on mitochondrial morphology in the sham and CLP groups remain unclear. We should point out, however, that although Parkin overexpression altered the mitochondrial morphology in skeletal muscles, none of the parameters related to mitochondrial dynamics that we investigated were affected by Parkin overexpression. Indeed, no significant impact of Parkin overexpression on the expression and protein content of MFN2, OPA1 and DRP1 was evident. Furthermore, Parkin overexpression had no effect on DRP1 phosphorylation on Ser^616^, suggesting that there was no change in DRP1 activity. Further studies are therefore required to identify the mechanisms underlying the differential impact of Parkin overexpression on skeletal muscle mitochondrial morphology in healthy and septic animals.

In the current study, we report data indicating that autophagy was induced in septic skeletal muscles, as evidenced by the increased expression of several autophagy-related genes, including *Lc3b*, *Gabarapl1*, and *Sqstm1*, and by the increase in the LC3B-II/LC3B-I ratio in the muscles of CLP mice. These findings are in agreement with previous reports, which documented increased muscle autophagy in various models of sepsis [20,30,53]. An interesting finding in our study is that Parkin overexpression had no effects on the expression of autophagy-related genes and the LC3B-II/LC3B-I ratios in the sham and CLP groups. Given the key role that Parkin plays in the recycling of dysfunctional mitochondria by autophagosomes [23,50], our results indicate that basal and activated autophagy levels in normal and septic muscles, respectively, were sufficient to deal with increased mitophagy in muscles overexpressing Parkin. We also observed that BNIP3 mRNA and protein levels increased significantly in septic skeletal muscles, and that this induction was not influenced by Parkin overexpression. The BNIP3 protein localizes to the mitochondria and promotes PINK1-Parkin-independent mitophagy by interacting with the LC3 protein, resulting in the recruitment of autophagosomes to damaged mitochondria [54]. The lack of changes in BNIP3 levels in response to Parkin overexpression suggests that BNIP3-mediated mitophagy functions in an independent fashion to that of the PINK1-Parkin pathway. It is worth mentioning that the present study suffers from several limitations. First, we did not directly assess whether Parkin overexpression actually translates into increased mitophagic flux. Although it was recently reported that Parkin overexpression is sufficient to trigger higher mitochondrial clearance in cardiomyocytes [55], further studies should investigate whether Parkin overexpression is sufficient to increase mitophagy in healthy and septic skeletal muscles. Another important limitation arises from the fact that muscle contractility was not assessed in the present study. Further studies are therefore required to define whether Parkin overexpression can attenuate sepsis-induced skeletal muscle weakness.

## 5. Conclusions

The present study provides evidence that Parkin overexpression attenuates sepsis-induced myofiber atrophy and prevents sepsis-induced changes in mitochondrial morphology and protein contents. These findings suggest that targeting mitophagy may represent a promising therapeutic strategy to attenuate sepsis-induced skeletal muscle wasting.

## Supplementary Materials

The following are available online at https://www.mdpi.com/2073-4409/9/6/1454/s1: Figure S1, Construction of the AAV1 designed to overexpress Parkin; Figure S2, The impact of sepsis and Parkin overexpression on the content and phosphorylation levels of proteins regulating protein synthesis; Figure S3, Additional TEM images; Table S1, List of antibodies; Table S2, List of qPCR primers.
