# Supplementary material for: Parkin Overexpression Attenuates Sepsis-Induced Muscle Wasting
Source: Cells. 2020 Jun 11;9(6):1454. doi: 10.3390/cells9061454 (PMC7349807; doi:10.3390/cells9061454)
Supplement: Supplementary file 1 [file cells-09-01454-s001.pdf]

Supplemental Materials

Figure S1. Construction of our AAV1 designed to overexpress Parkin

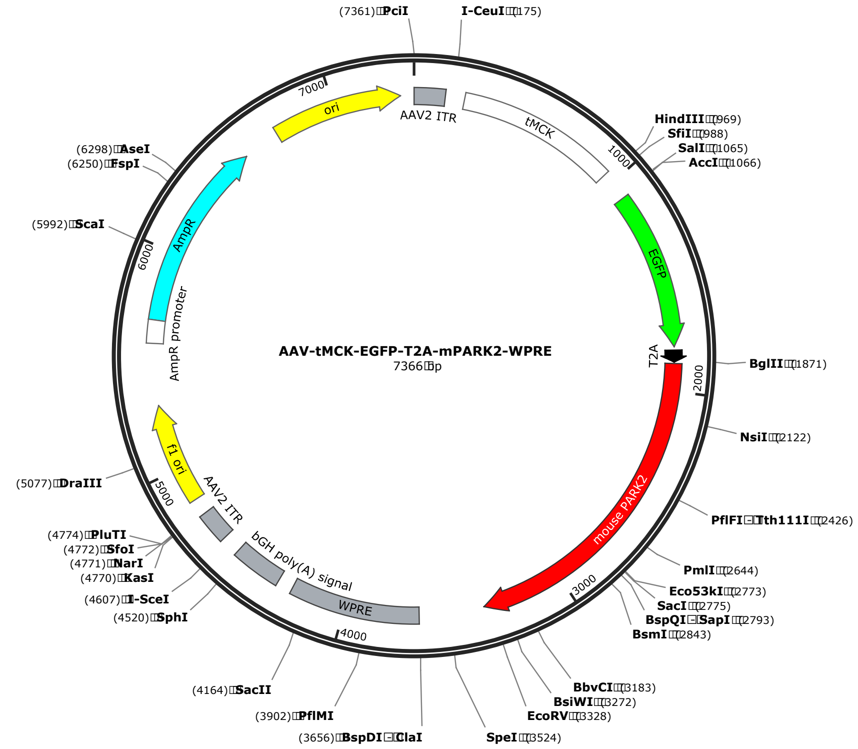

Figure S2. The impact of sepsis and Parkin overexpression on the content and phosphorylation levels of proteins regulating protein synthesis. Immunoblot detection and quantification of p-AKT (Ser473), AKT, p4EBP1(Thr37/46) and 4EBP1. Ponceau stains were used as loading controls. Data are presented as mean ± SEM. (n= 6-9/group).

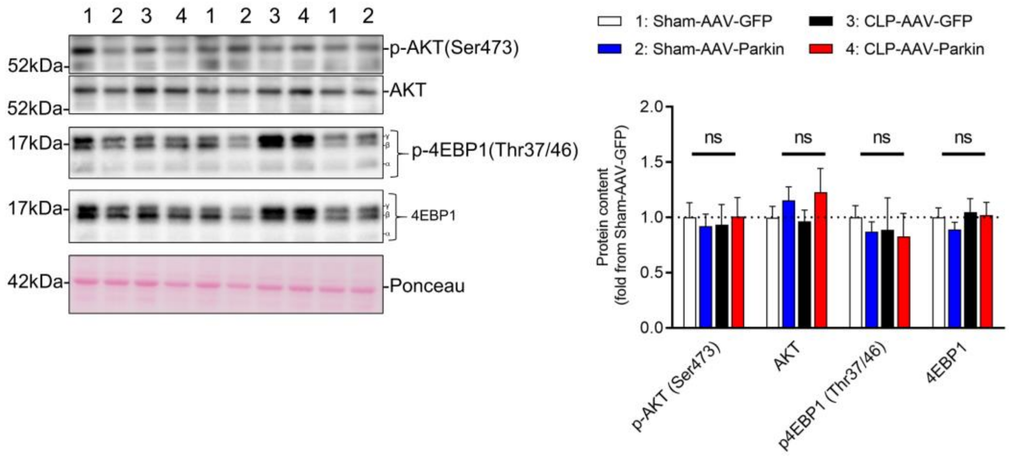

Figure S3. Additional TEM images.

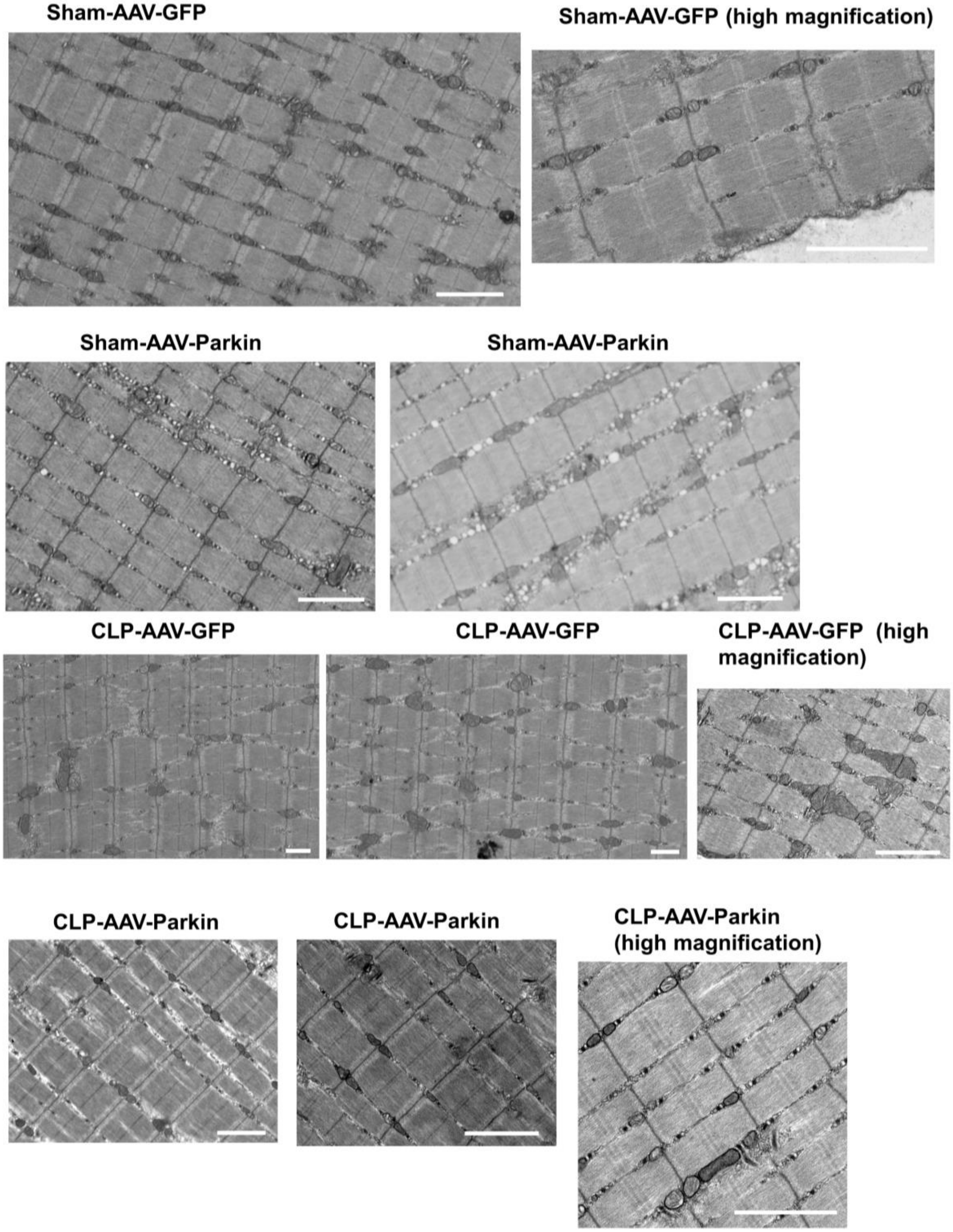

Representative longitudinal TEM images that were used to assess the impact of sepsis and Parkin overexpression on mitochondrial morphology. Scale bar: 2μm.

Table S1. List of antibodies

Protein	Dilution	Product no.	Source
BNIP3	1/1000	B7931	Sigma-Aldrich
DRP1	1/1000	# 611113	BD Biosciences
p-DRP1 (Ser616)	1/1000	#3455	Cell Signaling Technology
OPA1	1/1000	#42364	Abcam
MFN2	1/1000	#50843	Abcam
LC3A/B	1/1000	#12741	Cell Signaling Technology
PARKIN	1/1000	sc-32282	Santa Cruz
SQSTM1 (p62)	1/1000	clone 2C11	Novus Biologicals
OXPHOS	1/1000	ab110413	Abcam / MitoSiences
GAPDH (14C10)	1/5000	#2118	Cell Signaling Technology
VADC1/Porin	1/1000	ab14734	Abcam
p-Akt (Ser473)	1/1000	#9271	Cell Signaling Technology
Akt	1/1000	#9272	Cell Signaling Technology
p-4EBP1 (Thr37/46)	1/1000	#9459	Cell Signaling Technology
4EBP1	1/1000	#9644	Cell Signaling Technology

Table S2. List of qPCR primers

	**Forward primer (5'-3')**	**Reverse primer (3'-5')**
β-Actin	AACCGTGAAAAGATGACCCAG	CACAGCCTGGATGGCTACGTA
18S	TGCGGTTTAGCGTCGGTGTC	CCAAGTGGCCAAAGCGTA
Cyclophylin B	GCGTCTCTTCGAGCTGTTT	CTGGCACATGAATCCTGGAA
Bax	GGAGATGAACTGGATAGCAATATGG	GTTTGCTAGCAAAGTAGAAGAGGGC
Bid	CACAACATCCAGCCCACACT	CTCCATGTCTCTGGGGAAGG
Bim	GCAATGGCTTCCATACGACA	TTGCAAACACCCTCCTTGTG
Bcl2	CATGTGTGTGGAGAGCGTCA	ATCCCAGCCTCCGTTATCCT
BclXL	TGGGGAGCAACATTCATCAG	AGCTCCTCCTCCATCCCTTC
Mfn1	ATGGCAGAAACGGTATCTCCA	CTCGGATGCTATTCGATCAAGTT
Mfn2	GCTCAGGAGCAGCGGGTTTA	TGTGGACACCTGCCTTTCCA
Opa1	CAGAGGATGGTGCTCGTGGA	TCCGTCTTGGATGCACAGGA
Fis1	TGTCCAAGAGCACGCAATTTG	CCTCGCACATACTTTAGAGCCTT
Drp1	CCTCAGATCGTCGTAGTGGGA	GTTCCTCTGGGAAGAAGGTCC
Lc3b	CGATACAAGGGGGAGAAGCA	ACTTCGGAGATGGGAGTGGA
Bnip3	TTCCACTAGCACCTTCTGATGA	GAACACCGCATTTACAGAACAA
p62/SQSTM1	GCACCTGTCTGAGGGCTTCT	GCTCCAGTTTCCTGGTGGAC
Park2	TCTTCCAGTGTAACCACCGTC	GGCAGGGAGTAGCCAAGTT
Gabarapl1	GAGGACCACCCCTTCG	CGGAGGGCACAAGGTACTTC
Fbxo32	TGGGTGTATCGGATGGAGAC	TCAGCCTCTGCATGATGTTC
Trim63	TGCTTGGCACTTGAGAGGAA	AGAAGCTGGGCTTCATCGAG
Tfam	GCACCCTGCAGAGTGTTCAA	CGCCCAGGCCTCTACCTT
Nrf1	CGCTCATCCAGGTTGGTACA	TCCATCAGCCACAGCAGAGT
Nrf2	CTCCCAGGTTGCCCACATTC	TCAGTCATGGCTGCCTCCAG
Sirt1	GGATGATATGACAAACCCCAG	ACAGGAGACAGAAACCCCAG
Sirt3	CCCCGCTGCCCTGTCTGATC	TGATGAGCAGTCGGGGCACTG
Pgc1-α	TTGCTAGCGGTCCTCACAGA	GTCAGGCATGGAGGAAGGAC
Pgc1-β	GACCCCTTCAAGCCAGACAC	TGAGACTGGTTGGGTTGTGG
